# Sedentary Behaviour Profiling of Office Workers: A Sensitivity Analysis of Sedentary Cut-Points

**DOI:** 10.3390/s16010022

**Published:** 2015-12-25

**Authors:** Simone T. Boerema, Gerard B. Essink, Thijs M. Tönis, Lex van Velsen, Hermie J. Hermens

**Affiliations:** 1 Telemedicine Group, Roessingh Research and Development, P.O. Box 310, 7500 AH, Enschede, The Netherlands; g.b.essink@student.utwente.nl (G.B.E.); t.tonis@rrd.nl (T.M.T.); l.vanvelsen@rrd.nl (L.V.); h.hermens@rrd.nl (H.J.H.); 2 Telemedicine Group, Faculty of Electrical Engineering, Mathematics and Computer Science, University of Twente, P.O. Box 217, 7500 AE, Enschede, The Netherlands

**Keywords:** sedentary behavior, activity sensor, accelerometer, cut-point, activity pattern, office workers, laboratory trial, field trial

## Abstract

Measuring sedentary behaviour and physical activity with wearable sensors provides detailed information on activity patterns and can serve health interventions. At the basis of activity analysis stands the ability to distinguish sedentary from active time. As there is no consensus regarding the optimal cut-point for classifying sedentary behaviour, we studied the consequences of using different cut-points for this type of analysis. We conducted a battery of sitting and walking activities with 14 office workers, wearing the Promove 3D activity sensor to determine the optimal cut-point (in counts per minute (m·s^−2^)) for classifying sedentary behaviour. Then, 27 office workers wore the sensor for five days. We evaluated the sensitivity of five sedentary pattern measures for various sedentary cut-points and found an optimal cut-point for sedentary behaviour of 1660 × 10^−3^ m·s^−2^. Total sedentary time was not sensitive to cut-point changes within ±10% of this optimal cut-point; other sedentary pattern measures were not sensitive to changes within the ±20% interval. The results from studies analyzing sedentary patterns, using different cut-points, can be compared within these boundaries. Furthermore, commercial, hip-worn activity trackers can implement feedback and interventions on sedentary behaviour patterns, using these cut-points.

## 1. Introduction

High amounts of sedentary behaviour—sitting or reclining—are associated with increased risk of morbidity and mortality, independently of the level of moderate- to vigorous-intensity physical activity [1,2,3]. Moreover, there is little association between the time spent sitting and the time spent physically active in the course of a day [4], meaning that an individual can be simultaneously very sedentary and sufficiently physically active at a moderate- to vigorous intensity level.

A growing body of evidence indicates that not only the total sitting time, but also the pattern of accumulation of sitting time seems to mitigate health risks (such as a direct effect on metabolism, bone mineral content, and vascular health), independent of the total sitting time [4,5,6]. However, the more recent focus on sedentary behaviour has not yet resulted in general guidelines regarding which aspects of sedentary behaviour are most relevant to study.

We can measure the pattern of accumulation of sedentary time with wearable accelerometers on a minute to minute base, over longer periods of time. These sensors are primarily designed to measure intensity of physical activity, and not to distinguish postures such as sitting and reclining from standing and walking. This means that they cannot capture the full definition of sedentary behaviour being “any waking behaviour characterized by an energy expenditure of ≤1.5 metabolic equivalents, while in a sitting or reclining posture” [7]. However, these acceleration intensity based sensors, such as the Actigraph, are often used in sedentary research in which a cut-point is applied to classify a minute as being sedentary or active, based on the average acceleration intensity of that minute.

With the shift of focus from total sedentary time towards the pattern of accumulation of sedentary time, the number of measures capturing aspects of these patterns have also increased. These measures often focus on the duration of sitting periods (bout lengths) during the day. Examples of these bout measures, are the mean and median bout lengths, and more complex bout length distributions, such as the W_50%_ [8], the bout duration above and below which half of all sedentary time is accrued, and the Gini index [9], describing the inequality of bout lengths.

In literature reported sedentary behaviour measures are often based on different methods of classifying sedentary vs active behaviours and only a few studies have researched the effect of these various methods to the outcome parameters of sedentary behaviour [10,11,12]. Lyden *et al.* [12] found different total sedentary time and number of sedentary bouts for the 2 cut-points they studied (100 and 150 counts per minute), with increasing accuracy and precision as the cut-point increased. They conclude that the accuracy in estimating sedentary time and the number of bouts depended, among others, on the cut-point used to distinguish sedentary time, and the behavior of the sample population. However, by only comparing the effect of two different cut-points, it is unclear how strong the effect of the cut-point is, and in what range this effect is apparent. A sensitivity analysis with various cut-points will tell us whether or not it is possible to compare pattern measures of sedentary behaviour of various studies, while reducing the chance of building upon incorrect assumptions regarding sedentary behaviour, when used in interventions towards healthier lifestyles.

In this paper, we study how sensitive sedentary pattern measures are to various cut-points for sedentary behaviour. For this, we first determine the optimal cut-point for sedentary behaviour by means of direct observation of various activities in a laboratory setting (part A). Then, we vary around this optimal cut-point to perform a cut-point sensitivity analysis on a number of sedentary pattern measures of free living office workers (part B). This sensitivity analysis will show how sensitive sedentary pattern measures are to changes in the cut-point applied in accelerometer based sensors. We will conclude this article with the implications resulting from this sensitivity analysis for determining cut-points and the comparability of literature.

## 2. Method

### 2.1. A—Determining The Optimal Cut-Point for Sedentary Behaviour

#### 2.1.1. Protocol

Fourteen healthy office workers (average age 31.0 ± 8.7; 6 men/8 women) without physical complaints were asked to perform a battery of tasks related to office work in a laboratory setting via a snow ball sample. The task battery consisted of the following active and sedentary tasks:

a) Sitting for 2 min on a wheeled office chair;
b) Doing deskwork for 4 min (including reading, taking a book from the shelf and typing);
c) Sitting “restless” on a chair for 2 min (*i.e.*, to be active, while being seated);
d) Rising from a chair (one sit-stand transition) followed by 2 min of walking;
e) Walking through a corridor;
f) Standing still for 2 min.

During these tasks, participants wore the Promove 3D activity sensor (Inertia Technology, Enschede, The Netherlands) on the most lateral position, clipped to their waist belt [13]. Alongside this, a trained researcher annotated the start and stop time of each task on a dedicated smartphone application by direct observation. This application was synchronized with the data from the activity sensor. At the start of each session, participants were provided with an information letter and informed consent form.

#### 2.1.2. Data Analysis

The activity sensor samples the accelerations in three dimensions at 40 Hz and calculates per minute an average sum of the Integral of the Modulus of Accelerations (IMA) according to Equation (1) as described in Boerema *et al.* [13]. This value per minute is in metric units (m·s^−2^). However, for readability and to adhere to jargon in this research field, the values will be referred to as counts per minute (cpm) without its unit.

(1) $$IMA=\frac{1}{f_{s}T}\overset{n_{0}+f_{s}T}{\underset{n=\;n_{0}}{\sum }}\left|a_{x}\left[n\right]\right|+\left|a_{y}\left[n\right]\right|+\left|a_{z}\left[n\right]\right|$$

The optimal cut-point was defined as the point where sensitivity and specificity of sedentary tasks were equal. With tasks a, b, c and f being sedentary and tasks (based on their intensity level) d and e as active tasks. Sensitivity and specificity were calculated for various cut-points on a minute-by-minute base, see Equations (2) and (3), resulting in an ROC curve (Receiver Operating Characteristic) with sensitivity against the 1-specificity for various cut-points.

(2) $$Sensitivity=\frac{True\;Positives}{True\;Positives+False\;Negatives}$$

(3) $$Specificity=\frac{True\;Negatives}{True\;Negatives+False\;Positives}$$

### 2.2. B—Analyzing the Sensitivity of Sedentary Behaviour Patterns

#### 2.2.1. Protocol

Twenty-seven healthy office workers (average age 37.9 ± 13.5; 12 men/15 women) without physical complaints were asked to wear the activity sensor during waking hours of five working days. This population includes the 14 office workers who also participated in the first trial. The sensor produced the same IMA values per minute as in the laboratory trial. Again, participants were provided with an information letter and informed consent form at the start of the evaluation.

#### 2.2.2. Data Analysis

Each IMA value was classified as either sedentary or active using a cut-point value. For the sensitivity analysis this cut-point varied up to ±50% of the optimal sedentary cut-point as determined in the laboratory study. For each cut-point the following sedentary behaviour measures were calculated per person over the 5 day period:

- *Total sedentary time*: as percentage of total wear time;
- *Sedentary bout length*: as mean, median and W_50%_, in minutes. With the *W_50%_* being the bout length above and below which half of all sedentary time is accrued, calculated according to Chastin *et al.* [8];
- *Sedentary bout length distribution*: Gini index, between 0 and 1. With the Gini index describing the inequality of bout lengths, calculated according to Chastin *et al.* [9].

Statistical significant differences were tested with analysis of variances (ANOVA), with significance level of 0.05, when Levene’s test for homogeneity of variances was non-significant. Post-hoc tests were conducted if the ANOVA yielded a significant result, with a Bonferroni post-hoc test. Here, we applied a significance level of 0.01 because of the high number of post-hoc tests conducted. When Levene’s test was significant, we used a corrected version of the F-ratio: Welch’s F, after which we conducted the same post-hoc test.

## 3. Results and Discussion

### 3.1. A—The Optimal Cut-Point For Sedentary Behaviour

**Figure 1:**
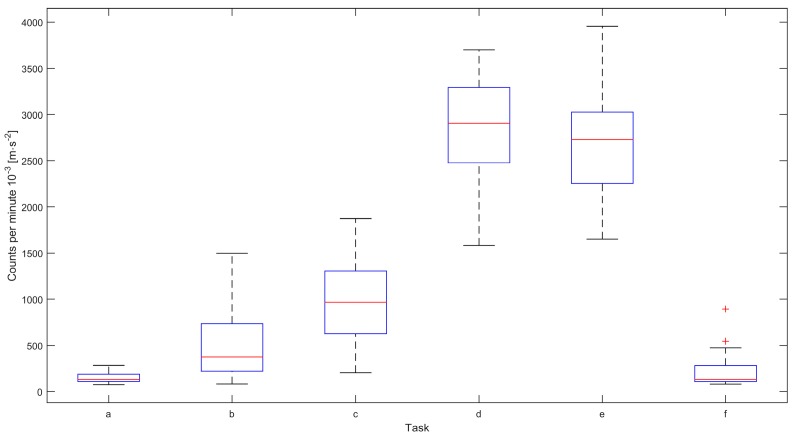
Boxplots of count per minute for the active and sedentary tasks (*n* = 14). (**a**) sitting; (**b**) doing deskwork; (**c**) sitting “restless”; (**d**) rising from a chair and walking; (**e**) walking; and (**f**) standing still.

The average counts per minute for the sedentary tasks (a, b, c) was 531 (± 468) × 10^−3^ m·s^−2^, and for the active tasks (d and e) 2770 (± 568) × 10^−3^ m·s^−2^. The average counts per minute while standing still (task f) was 219 (± 182) × 10^−3^ m·s^−2^, as shown in Figure 1.

**Figure 2:**
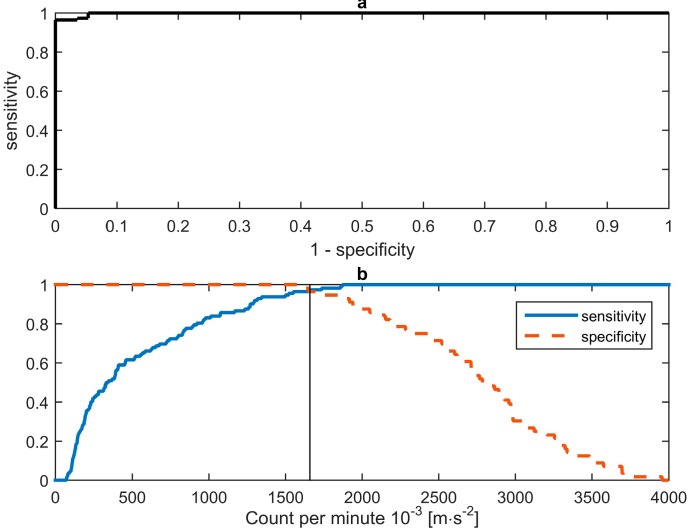
(**a**) ROC curve in counts per minute (sensitivity *vs*. 1-specificity), based on tasks a–e. Area under the curve is 0.9982. (**b**) Sensitivity and specificity vs. cut-point values. The curves intersect at: 1660 × 10^−3^ m·s^−2^.

Task f—standing still—was within the same range of counts per minute as the sitting tasks, and could therefore not be classified as an active task based on a single cut-point. If task f was included in the ROC curve, this resulted in an area under the curve of 0.7548, indicating a rather poor performance. As it is known that standing still cannot be classified, based on counts per minute, we excluded task f from the ROC analysis as commonly done in accelerometer intensity based studies (e.g., by Kozey *et al.* [10] and Aguila-Farías *et al.* [11]). Excluding task f “standing still” from the ROC analysis resulted in a sensitivity and a specificity curve crossing at 96.43%, corresponding to the optimal cut-point of 1660 × 10^−3^ m·s^−2^, see Figure 2b. The area under the curve of the ROC was 0.9982, indicating an excellent performance, see Figure 2a.

### 3.2. B—Sensitivity of Sedentary Behaviour Patterns

In total, 137 days were measured; on average 5.07 days per subject, of which two subjects wore the sensor for only 4 days. The mean wear time per day was 13 h 18 min ± 2 h 33 min. Sedentary time, bout lengths and the Gini index were calculated for various cut-points as shown in Table 1.

**Table 1:** Overview of sedentary pattern measures for various cut-points.

Cut-Point		Total Sedentary Time (%)	Sedentary Bout Length (min)			GINI Index [0–1]
(% ^a^)	(cpm)		*Mean*	*Median*	*W_50%_*	
50%	830	76.05 ^b^	13.51 ^b^	4.52 ^b^	39.67 ^b^	0.66
80%	1328	82.58 ^b^	15.64	4.56	48.11	0.67
90%	1494	84.16	16.41	4.81	52.04	0.67
95%	1577	84.92	16.83	4.94	53.41	0.67
100%	1660	85.66	17.34	5.09	54.78	0.67
105%	1743	86.40	17.92	5.35	56.07	0.67
110%	1826	87.09	18.39	5.61	58.15	0.67
120%	1992	88.42 ^b^	19.82	6.02	60.93	0.67
150%	2490	91.90 ^b^	26.84 ^b^	8.96 ^b^	76.96 ^b^	0.66

^a^ Percentage of the optimal cut-point of 1660 counts per minute (cpm) in 10^−3^ (m·s^−2^); ^b^ Significant different from the value at cut-point 1660 × 10^−3^ m·s^−2^, with α = 0.05.

#### 3.2.1. Total Sedentary Time

**Figure 3:**
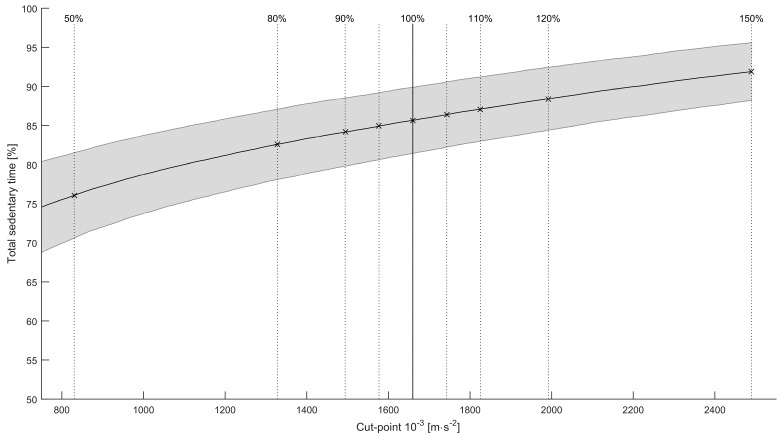
Mean total sedentary time as percentage of wear time for various cut-points. The shaded area is the standard deviation. Vertical lines indicate the various thresholds, with the solid line being the optimal cut-point.

Total sedentary time was, on average, 85.66% ± 4.22% for the optimal cut-point, see Figure 3 for a visual representation. Levene’s test for homogeneity of variances was non-significant. There was a significant effect of cut-point on total sedentary time, F(8, 234) = 27.30, *p* < 0.001. Post-hoc tests showed that the total sedentary time did not change significantly within the −10% and +10% threshold intervals. We did find significant differences outside this range (where −50% differed significantly from all other cut-points; −20% from +10%, +20%, +50%; and +50% from all but +20%).

#### 3.2.2. Sedentary Bout Lengths

The sedentary bout length distribution has a skewed distribution, with a mean duration of 17.3 ± 3.9 min and median duration of 5.1 ± 2.0 min when applying the optimal cut-point. With an increasing cut-point, the mean and median bout length, as well as their standard deviations increase. Since Levene’s test was significant for the mean and median sedentary bout length, we calculated Welch’s adjusted F ratio for both ANOVA tests.

There was a significant effect of cut-point on the mean sedentary bout length, Welch’s F(8, 97.33) = 8.85, *p* < 0.001. Post-hoc tests showed that the mean bout length did not change significantly within the −20% and +20% threshold intervals. We did find significant differences outside this range (where −50% differed significantly from +10% and above; and +50% differed significantly from all other cut-points).

There was also a significant effect of cut-point on the median sedentary bout length, Welch’s F(8, 97.14) = 3.50, *p* = 0.001). Post-hoc tests showed that the median bout length did not change significantly within the −20% and +20% threshold intervals. We did find significant differences outside this range (where +50% differed significantly from all other cut-points).

The W_50%_ measure (the bout duration above and below which half of all sedentary time is accrued), shows a larger standard deviation for the group than the mean and median bout lengths. Levene’s test for homogeneity of variances was non-significant. There was a significant effect of cut-point on W_50%_, F(8, 234) = 2.86, *p* = 0.005. Post-hoc tests showed that the W_50%_ did not change significantly within the −20% and +20% threshold intervals. We did find significant differences outside this range (where −50% differed significantly from +50%).

**Figure 4:**
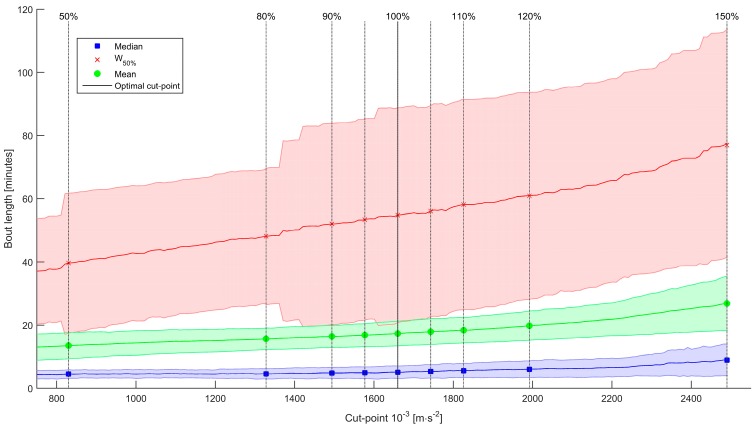
Bout length variables for various cut-points. Blue: Median bout length; green: Mean bout length; red: W_50%_ bout length. Shaded areas are the standard deviations.

This indicates that the mean, median and W_50%_ bout length measures are not sensitive to changes within the ±20% interval of the optimal cut-point, as shown in Figure 4.

#### 3.2.3. Sedentary Bout Distribution

**Figure 5:**
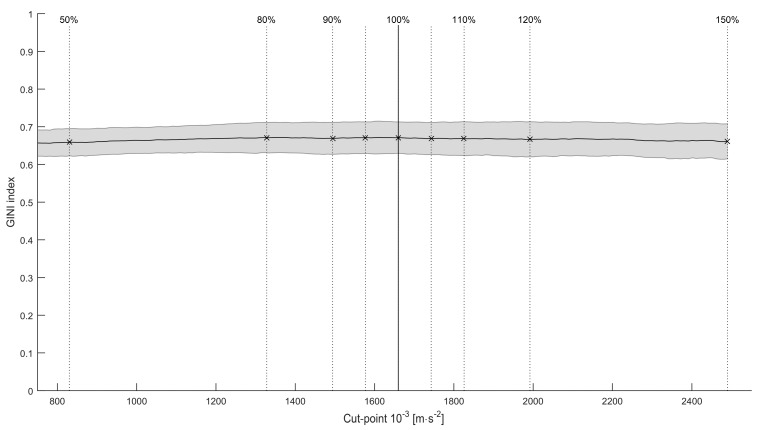
Mean Gini index for various cut-points. The shaded area is the standard deviation. Vertical lines indicate the various thresholds, with the solid line being the optimal cut-point.

The Gini index is very stable over the full range of cut-points of ±50% of the optimal cut-point, with a mean of 0.67 ± 0.04 at the optimal cut-point. Levene’s test for homogeneity of variances was non-significant. Moreover, there was no significant effect of cut-point on the Gini index, F(8, 234) = 0.30, p = 0.967, indicating that the Gini index is not sensitive to changes within the ±50% interval of the cut-point, as show in Figure 5.

## 4. Discussion

In this study we have shown that sedentary pattern measures of daily living of office workers showed relatively low sensitivity to changes in the cut-point for sedentary behaviour. This makes 3D accelerometry very suitable for sedentary pattern analysis, without dedicated calibration studies. Some of these measures were more sensitive than others. The percentage of sedentary time was the most sensitive parameter, the mean, median bout length and W_50%_ were less sensitive and the Gini index was the least sensitive, showing no significant change within the ±50% interval that was studied around the optimal cut-point.

The results of this study indicate that sedentary pattern measures that are applied and reported in literature are comparable if they are measured by an acceleration based activity sensor with a cut-point for sedentary behaviour defined in a likewise manner. This means that the cut-point is defined for sedentary behaviours versus active behaviours, in which “standing still” is considered to be an sedentary behaviour, which is a commonly accepted approach [4,10,11,12]. Based on our findings, these sedentary pattern measures are comparable if they are based on slight deviations from the optimal cut-point to distinguish active and sedentary behaviours, and this allowed deviation depends on the specific pattern measures, ranging from ±10% for total sedentary time, up to at least ±50% for the Gini index.

The calibration study described in this paper has shown that the counts per minute measured during the office related active and sedentary behaviours show almost no overlap, except for the behaviour “standing still”. This behaviour has an even lower acceleration intensity than sitting restless on a chair, thereby making it impossible to distinguish these two activities based on only the mean counts per minute. Other studies have shown that it is possible to classify specific behaviours based on a single wearable sensor. However, they need more detailed sensor information than one value per minute, such as high frequency data or additional sensors such as gyroscopes [14]. These more complex sensing and analysis methods often need calibration for groups or even individual subjects, which is hindering the comparability of reported behaviour patterns.

Moreover, it strongly depends on the research questions if ‘misclassification’ of standing is a problem or not. Chau *et al.* [15] showed that office workers were standing about 45 min ± 28 min per day, while sitting for 5 h 47 min ± 59 min per day. In this study sample, standing would only comprise 11% of the total sedentary time measured by the sensor. The vast number of studies using the ActiGraph and alike sensors, show that standing time classified as sedentary is a generally accepted limitation of the sensing method.

### Limitations

The sensitivity of the sedentary pattern measures described in this study, might be dependable on the limited number of type of activities in the free-living dataset. Because we focused on office workers, and predominantly during working hours, there were only a limited number of types of activities measured. These activities were predominantly sitting, walking, standing, and commuting by car or bike. And it is unknown what the contribution is to the sensitivity of the pattern measures with the current amount of the activity ”standing still”.

Changes in behaviour can be reflected differently in various sedentary patterns measures. Lyden *et al.* [12] did an intervention study in which office workers were asked to reduce and break up their sedentary time by replacing “sitting time” to “standing still” time at a sit-to-stand desk. Their definition of sedentary time included “standing still” and the change in behaviour resulted, therefore, in a larger overestimation of number of sedentary bouts, while the accuracy of the number of breaks per sedentary hour improved. These opposite effects on sedentary pattern measures with changes in the behaviour are strongly affected by the applied definition of sedentary behaviour and can also affect estimates of total sitting time and bout lengths described in the present study. However, our findings are valid for office workers when only including sitting time.

## 5. Conclusions

Both our findings on the sensitivity of sedentary patterns to various cut points, and our finding that previous studies with different cut-points can be compared within certain boundaries, opens avenues for more focused research in sedentary behavior patterns and in creating an in-depth understanding of habitual physical activity rhythms of sedentary and active periods. Understanding these rhythms and predicting active and sedentary behaviour clears the way for new innovative physical activity interventions towards healthy behaviour.

Additionally, it is valuable to investigate the quality of the full range of sedentary pattern measures described in literature, such as number of bouts [16], breaks per sedentary hour [17], and sequences of activity-rest periods [18]. In this paper we discussed five pattern measures and their sensitivity to various cut-points, but there are many more sedentary pattern measures described in literature. These are tested with various populations on their ability to capture the specific behaviour patterns of populations and their variability within groups. As in physical activity research has been done on reported intensity levels and bout lengths, sedentary behaviour researchers should work towards an overview of the best measures for specific research questions with indications of their strengths and weaknesses. This should help the field in applying sedentary information in clinical practice and thereby further maturing the research field.

Finally, commercially hip-worn activity trackers, like those developed by Fitbit, Misfit and Jawbone, can also benefit from the findings of this study. Their sensing methods are often accelerometer intensity based, which is the same method as is investigated in this study. Our findings, therefore, can also serve as valuable input for the development of feedback and intervention protocols focused on sedentary behavior for these sensors. This way, these sensors can enter a new domain within activity tracking by means of consumables, and provide their customers with an overview of their daily sedentary behaviour additional to the number of steps and burned calories.
